# Research on Properties of PBAT/CaCO_3_ Composite Films Modified with Titanate Coupling Agent

**DOI:** 10.3390/polym15102379

**Published:** 2023-05-19

**Authors:** Zhekun Liu, Fantao Meng, Xianggang Tang, Chengzhuang Su, Qinglin Mu, Guannan Ju

**Affiliations:** School of Materials Science and Engineering, Shandong University of Technology, Zibo 255000, China; liuzk1101@163.com (Z.L.); 15836877753@163.com (X.T.); sumuo1119@163.com (C.S.); 17864301319@163.com (Q.M.); ju@sdut.edu.cn (G.J.)

**Keywords:** PBAT, CaCO_3_ particles, titanate coupling agent, compatibility

## Abstract

High cost, low crystallinity, and low-melt strength limit the market application of the biodegradable material poly (butylene adipate-co-terephthalate) (PBAT), which has become a major obstacle to the promotion of PBAT products. Herein, with PBAT as resin matrix and calcium carbonate (CaCO_3_) as filler, PBAT/CaCO_3_ composite films were designed and prepared with a twin-screw extruder and single-screw extrusion blow-molding machine designed, and the effects of particle size (1250 mesh, 2000 mesh), particle content (0–36%) and titanate coupling agent (TC) surface modification of CaCO_3_ on the properties of PBAT/CaCO_3_ composite film were investigated. The results showed that the size and content of CaCO_3_ particles had a significant effect on the tensile properties of the composites. The addition of unmodified CaCO_3_ decreased the tensile properties of the composites by more than 30%. TC-modified CaCO_3_ improved the overall performance of PBAT/CaCO_3_ composite films. The thermal analysis showed that the addition of titanate coupling agent 201 (TC-2) increased the decomposition temperature of CaCO_3_ from 533.9 °C to 566.1 °C, thereby enhancing the thermal stability of the material. Due to the heterogeneous nucleation of CaCO_3_, the addition of modified CaCO_3_ raised the crystallization temperature of the film from 97.51 °C to 99.67 °C and increased the degree of crystallization from 7.09% to 14.83%. The tensile property test results showed that the film reached the maximum tensile strength of 20.55 MPa with the addition of TC-2 at 1%. The results of contact angle, water absorption, and water vapor transmission performance tests showed that TC-2 modified CaCO_3_ increased the water contact angle of the composite film from 85.7° to 94.6° and decreased the water absorption from 13% to 1%. When the additional amount of TC-2 was 1%, the water vapor transmission rate of the composites was reduced by 27.99%, and the water vapor permeability coefficient was reduced by 43.19%.

## 1. Introduction

The use of traditional plastics has caused serious environmental pollution problems [1]. In recent years, with the rapid development of industries, such as express delivery and takeaway, the production capacity in the field of plastic packaging has increased year by year, and the use of traditional plastic packaging products has become more widespread, with the consequent economic and social problems becoming increasingly serious [2]. As a result, several countries around the world have enacted corresponding laws and regulations to encourage the use of biodegradable materials, which has promoted research into easily recyclable, easily degradable blends and compounds [3,4]. However, most biodegradable plastics are three–five times more expensive than traditional plastics, making them difficult to use widely. Therefore, research on low-cost and high-performance degradable materials is an effective solution to the problem of “white pollution” [5].

Poly (butylene adipate-co-terephthalate) (PBAT) is a new biodegradable polyester synthesized by polycondensation of terephthalic acid, adipic acid, and dibutyl ester. It has both aliphatic and aromatic segments in its molecular structure, which gives it good toughness and rigidity and makes it suitable for blow-molding into a film for use in the plastic packaging industry. Therefore, it is considered the most suitable substitute for polyethylene packaging products [6,7]. Over the past few years, the growth of the take-out and delivery industries has greatly contributed to the use of plastic products in packaging. However, compared to conventional plastics, such as polyethylene (PE), PBAT has high production costs, poor mechanical properties, and low crystallinity, all of which limit its market application [8,9]. Therefore, it is important to select appropriate functional materials and plastic processing aids to modify PBAT to improve its comprehensive performance and reduce the application cost.

In order to overcome the above-mentioned drawbacks, many scholars have devoted themselves to the study of PBAT modification in the last five years. In order to facilitate the expansion of PBAT packaging products for market applications, many inorganic fillers and natural fibers have been incorporated into the PBAT matrix to reduce the production cost of the products, such as silica [10,11], talc [12,13], and so on. Many reinforcing fillers and other kinds of resins are incorporated into the PBAT matrix to improve the mechanical properties of the products, such as polylactic acid (PLA) [14,15], polyethylene [16,17], polypropylene (PP) [18,19], polyglycolic acid (PGA) [20,21], and so on. As one of the most widely used fillers in plastic modification, the price of CaCO_3_ is only 3%–5% of that of PBAT. The addition of CaCO_3_ to PBAT can not only reduce the production cost of PBAT products but also improve the mechanical properties of PBAT products [13,22,23,24].

The main influencing factors of CaCO_3_-reinforced composites are CaCO_3_ content, particle size, particle dispersion, and interfacial bonding effect between particles and matrix. It is found that the mechanical properties of CaCO_3_-reinforced resin matrix composites are generally worse than those of pure polymer materials because the compatibility between CaCO_3_ and polymer matrix is poor. With the increase in CaCO_3_ content, the particles are more likely to agglomerate in the composite system, resulting in uneven distribution of particles in the system and poor interfacial bonding, while the performance of the composite depends largely on the interfacial bonding effect between the reinforcing phase and the matrix [25,26,27]. In addition, the general CaCO_3_ powder will contain about 1% water. When it is blended with polyester, the water in CaCO_3_ will accelerate the degradation of polyester, thus reducing the mechanical properties of composite materials.

Modification of inorganic filler particle surfaces with surface modifiers is one of the most common and effective methods to suppress the particle agglomeration phenomenon and improve the poor compatibility between the matrix and reinforcing phases of composites [28]. Surface modifiers are broadly classified into two types according to the mechanism of action. One is a compatibility agent, which is a third component added to the system to improve the problem of poor compatibility of polymers during blending. Its function is to reduce the interfacial tension, increase the thickness of the interfacial phase and prevent the coalescence of the dispersed phase. It has no reactive groups, and no chemical reaction occurs during processing. It only relies on the diffusion of chain segments in the molecule or Van der Waals forces to increase the adhesion between components. Maleic anhydride, maleic anhydride graft copolymers, or block copolymers are the most applied compatibilizers and are widely used in the study of low-density polyethylene (LDPE)/CaCO_3_ and high-density polyethylene (HDPE)/CaCO_3_, PLA/CaCO_3_ systems [29,30,31]. The other is a coupling agent, which is an additive used to enhance the performance of the interface between the synthetic resin and inorganic filler or reinforcing material. Its function is to reduce the melt viscosity of synthetic resin during processing and improve the dispersion of inorganic filler. It consists of two parts. One part can react with inorganic fillers, and the other can react chemically with polymers or form hydrogen bonds. When the coupling agent is used to treat the surface of inorganic filler, one end of its molecular chain is wound or reacted with the molecular chain of the matrix resin and the other end of the inorganic filler is bonded to the surface, thus improving the compatibility between inorganic filler and matrix resin and improving the mechanical properties of composite materials [32]. Silane coupling agents, titanate coupling agents, and aluminate coupling agents are currently the most widespread coupling agents. According to the reaction mechanism, the silane coupling agent has a better coupling effect on the filling system containing polar groups or introducing polar groups, but it has no obvious effect on the nonpolar system and has a poor effect on the CaCO_3_-filled composite system [33]. When CaCO_3_ fills degradable polyester, TC is more suitable for modification. This is because CaCO_3_ and PBAT particles contain trace amounts of water, and TC can react with the hydroxyl groups of water molecules to form chemical bonds, which slow down the degradation of PBAT molecular chains while achieving the purpose of chemical coupling. Moreover, the molecule of TC contains long alkane groups, which can be entangled with the PBAT molecular chain, thus improving the compatibility between PBAT and CaCO_3_ and reducing the melt strength of composites during processing.

The research on filling traditional polymer materials, such as PE, styrene-butadiene-styrene (SBS), polyvinyl chloride (PVC), polyamide (PA), and acrylonitrile butadiene styrene (ABS) with CaCO_3_ particles modified by TC has been reported [34,35,36,37,38]. Until now, the research on the PBAT-filled modified CaCO_3_ has mainly focused on nano-CaCO_3_, and the coupling agents have mostly used silane coupling agents. However, because of the high specific surface area and surface energy of nano-CaCO_3_, the nano-CaCO_3_ added to the PBAT matrix is more easily agglomerated than the micron-sized CaCO_3_ particles, whose content is mostly below 15% [39,40,41].

In this study, surface modifier dilution spraying is used to investigate the effects of CaCO_3_ particle size (1250 and 2000 mesh), content (0–36 wt%), and surface modification (using different types and amounts of titanate coupling agents) on the tensile properties, melting temperature, crystallization temperature, crystallinity, thermal stability, hydrophilicity, water absorption, and water vapor permeation properties. So far, no studies have been reported on the properties of PBAT films filled with CaCO_3_ particles modified with different types of TC agents by the pretreatment method. Therefore, it is necessary to analyze the effects of these factors on the above properties of PBAT/CaCO_3_ films to provide theoretical and practical guidance to ensure the excellent properties of PBAT films.

## 2. Materials and Methods

### 2.1. Materials

PBAT is provided by Yingkou Kang Hui Petrochemical Co., Ltd. (Liaoning, China). CaCO_3_ powder (1250 mesh, 2000 mesh) is provided by Zibo Shuangfeng Chemical Co., Ltd. (Shandong, China). Titanate coupling agent 131 (abbreviated as TC-1) is provided by Dongguan Yisheng Technology Co., Ltd. (Guangdong, China). Titanate coupling agent 201 (abbreviated as TC-2) is provided by Lvwei New Materials Technology Co., Ltd. (Guangdong, China).

Chemical structure of TC-1: (CH_3_)_2_CHOTi [OC(O)(CH_2_)_14_CH(CH_3_)_2_]_3_.

Chemical structure of TC-2: (CH_3_)_2_CHOTi [OP(O)(OH)- OP(O)-(OC_8_H_17_)_2_]_3_.

### 2.2. Thermal Processing of Composite Materials

#### 2.2.1. CaCO_3_-Modified PBAT Films with Different Particle Sizes and Contents

Before the thermal processing, PBAT and CaCO_3_ were placed in an oven at 100 °C for 10 h in order to dry the moisture absorbed by the raw material in the air. Then, PBAT and CaCO_3_ were weighed according to the experimental formulation in Table 1, and 5 kg was weighed for each group. Then, after preliminary mixing, the materials were poured into a twin-screw extruder, melted and extruded, cooled by air, and granulated by a granulator. Co-blending extrusion was performed using a twin-screw extruder, and after cooling, the extruded samples were finally pelletized and dried. The ratio of extruder screw length to diameter was 50, and the speed was 30 rpm. The extruder was divided into 9 temperature zones, and the extrusion temperature ranged from 130 °C to 165 °C.

#### 2.2.2. PBAT Composite Filled with Modified CaCO_3_

Based on the test results of mechanical properties of unmodified CaCO_3_, we selected the sample containing 73% of PBAT and 27% of CaCO_3_ for the investigation of TC surface modification. The formula of PBAT composite filled with CaCO_3_ modified by TC is shown in Table 2. The percentage of TC added was relative to the amount of CaCO_3_, not the total mass of raw material. Firstly, PBAT and CaCO_3_ were placed in an oven at 100 °C for 10 h in order to dry the moisture absorbed by the raw material in the air. Then, TC was diluted into anhydrous ethanol in a ratio of 1:5 and sprayed onto the CaCO_3_ particles evenly. The CaCO_3_ powder was added into the high-speed mixer and stirred at 100 °C for 15 min to make the TC fully react with the hydroxyl groups on the surface of CaCO_3_ to form a polymer organic film, and the modified CaCO_3_ was obtained after cooling. Then, PBAT and modified CaCO_3_ were added to the twin-screw extruder for co-blending and extrusion with the same extruder settings as in Section 2.2.1.

### 2.3. Extrusion Blow-Molding Composite Film

The extruded composite pellets from the twin-screw extruder were added to the single-screw extrusion and blow-molding machine to form a film. The single-screw extruder had five temperature zones, and the extrusion blow-molding temperatures were 140 °C–165 °C. The extrusion rate was 15 Hz, and the film expansion rate was 2.5.

### 2.4. Testing and Characterization

#### 2.4.1. Tensile Properties

Before the tensile performance test, the prepared film samples were placed in the laboratory test environment for 24 h. The parameters were adjusted according to the test standard of GB/T1040.1-2018, and the films were tested for tensile performance using a microcomputer-regulated universal testing machine (INSTRON 5969, Instron Corporation, Norwood, MA, USA). During the test, the specimens were stretched at a speed of 20 mm/min, and the distance between fixtures was set to 25 mm. Five tests were performed on each group of samples, and the average value was taken according to the valid values.

#### 2.4.2. Hydrophilicity Testing

The contact angle is an important basis for characterizing the hydrophilicity of the material. According to the test standard of GB/T 30693-2014, the samples were placed at a temperature of (23 ± 2) °C and relative humidity of (50 ± 5)% for more than 40 h before the test. The water contact angle of the composite film was tested in a contact angle tester (Theta3040330, PROLONG FORTUNE INTERNATIONAL LIMITED, Hong Kong) under static conditions. The films were cut into strips of 50 mm × 300 mm, and a drop of deionized water was slowly dropped on the sample surface and captured by a camera, and then, the contact angle was determined by the image analysis software. Each set of specimens was tested 10 times to determine the contact angle of the film.

#### 2.4.3. Water Absorption Test

The water absorption of the composite films was determined according to ISO 62. The film samples were cut into 30 mm × 30 mm square pieces and dried in a vacuum oven at 85 °C until the weight was constant. The samples were then immersed in 50 mL of distilled water for 24 h at room temperature, wiped with filter paper to remove water from the surface of the specimens, and weighed to record the final weight. Each group of specimens was tested 10 times, and the effective values were averaged to obtain the water absorption rate of the film. The film water absorption *ω* was calculated according to Equation (1) [42]:

(1) $$\omega \;(\%)=100\;\times \;\frac{M_{2}-M_{1}}{M_{1}},\;$$

In Equation (1):

*ω*—the water absorption of the specimen, in %;
*M*_2_—the wet mass of the specimen after water absorption, in g;
*M*_1_—the dry mass of the specimen before water absorption, in g.

#### 2.4.4. Water Vapor Transmission Performance Test

The water vapor transmission properties of the films were tested according to GB/T 1037-2021 using a water vapor transmission tester (3/33MA, Mocon company, Minneapolis, MN, USA), and the samples with thicknesses of not more than 3 mm were cut into 10 cm × 10 cm squares and laid flat in a moisture permeable cup. The test was conducted 15 times at 90% of relative humidity and 38 °C with a weighing interval of 2 h. Three parallel samples were taken for each sample, and the water vapor transmission rate (WVTR) and water vapor transmission coefficient (WVP) were averaged.

#### 2.4.5. Microscopic Morphology Observation

The surface morphology of the films was investigated using a scanning electron microscope (SEM) (Quanta 250, FEI company, Hillsboro, OR, USA). The accelerating voltage was kept at 10 kV during the scanning process. The energy dispersive spectrometer (EDS) mapping analysis of the calcium element distribution of the system before and after modification was carried out using SEM with an accelerating voltage of 20 KV.

#### 2.4.6. Differential Scanning Calorimetry Analysis

The melting and crystallization behaviors of pure PBAT and PBAT/CaCO_3_ composites were analyzed by differential scanning calorimetry (DSC Q2000, TA instruments, New Castle, DE, USA). A 5 mg sample was heated from 20 °C to 200 °C at a constant rate of 10 °C/min under the N_2_ atmosphere and then held at this temperature for 3 min to eliminate the thermal history. Then, the samples were cooled to 20 °C at a constant rate of 10 °C/min. Finally, the samples cooled to 20 °C were reheated to 200 °C at a constant rate of 10 °C/min to characterize the melting and crystallization behavior of the material. The crystallinity Xc was calculated, as shown in Equation (2) [43]:

(2) $$X_{c}=\frac{\Delta H_{m}}{W_{f}\times \Delta H_{0}},\;$$

In Equation (2):

*X_c_*—the degree of crystallinity of PBAT, in %;
Δ*H_m_*—the enthalpy of melting of PBAT composites, in J/g;
Δ*H*_0_—the enthalpy of melting of PBAT, in 114 J/g;
*W_f_*—the mass fraction of PBAT accounted for in PBAT composites, in %.

#### 2.4.7. Thermogravimetric Analysis

Thermogravimetric analysis (TGA) was tested by a comprehensive thermal analyzer (SDT650, TA Instruments company, Newcastle, DE, USA). The samples were heated from 20 °C to 800 °C at a heating rate of 10 °C/min under the N_2_ atmosphere protection.

#### 2.4.8. Fourier Infrared Spectroscopy Analysis

The chemical structure of CaCO_3_ before and after modification was analyzed by infrared spectroscopy (Nicolet5700, Thermo Nicolet Corporation, Madison, WI, USA) in the wave number range of 400 cm^−1^–4000 cm^−1^.

## 3. Results and Discussion

### 3.1. Effect of CaCO_3_ Particle Size and Particle Content

PBAT/CaCO_3_ composite films are prepared by twin-screw melt blending and single-screw extrusion blow molding with CaCO_3_ particles of 1250 mesh and 2000 mesh. The effect of CaCO_3_ filling on the tensile properties of the film is tested by an electronic universal testing machine, and the effect of CaCO_3_ filling on the microstructure of the film is observed by scanning electron microscope.

#### 3.1.1. Effect of CaCO_3_ Particle Size and Content on Tensile Properties

**Figure 1:**
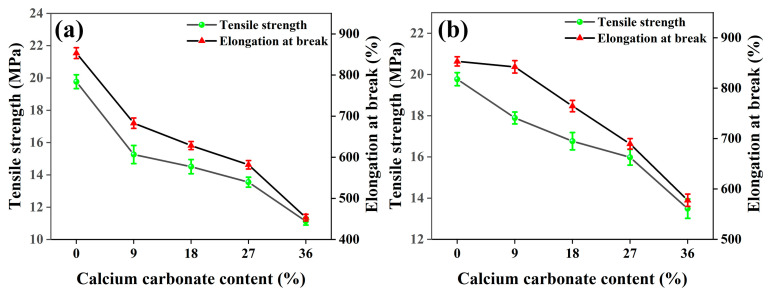
Effect of unmodified CaCO_3_ content on the tensile properties of PBAT/CaCO_3_ composite films (**a**) 1250 mesh (**b**) 2000 mesh.

The effects of 1250 mesh and 2000 mesh unmodified calcium carbonate filled with PBAT on the tensile properties of the composite films are shown in Figure 1a,b.

It can be seen from Figure 1a that with the gradual increase of 1250 mesh in CaCO_3_ content, the tensile strength and elongation at the break of the films show a decreasing trend. When the CaCO_3_ content increases from 0% to 36%, the tensile strength of the film decreases from 853.21% to 453.27%, a decrease of 46.87%. The test results show that the tensile properties of the composite films made of 1250-mesh unmodified CaCO_3_ directly filled with PBAT are significantly decreased compared with pure PBAT. As can be seen from Figure 1b, the tensile strength and elongation at the break of the films show a decreasing trend with the gradual increase in the CaCO_3_ content at 2000 mesh. When the CaCO_3_ content increases from 0% to 36%, the tensile strength and elongation at the break of the films show a decreasing trend. The tensile strength of the films decreases from 19.77 MPa to 13.49 MPa, a decrease of 31.77%, and the elongation at break decreases from 853.21% to 577.63%, a decrease of 32.30%. The test results show that the tensile properties of the composite films filled by 2000 mesh of unmodified CaCO_3_ also decrease significantly compared with pure PBAT, but the decrease is smaller than that of the composite prepared by 1250 mesh of unmodified CaCO_3_.

In addition, the composite films prepared from CaCO_3_ particles with smaller particle sizes have higher tensile strength and larger elongation at break when the CaCO_3_ addition amounts of the two-particle sizes are the same, which indicates that the size of CaCO_3_ particles has a significant effect on the mechanical properties of the composite films in the PBAT/CaCO_3_ system [44]. In general, in systems with inorganic filler-filled resins, an increase in inorganic filler particle size has a significant negative effect on the tensile properties of the composite [22]. On the contrary, as the inorganic filler particle size decreases, the specific surface area of the filler increases, more molecular chains can be adsorbed, and the aggregation tendency of the material increases. Therefore, the tensile properties of the films are better when the CaCO_3_ particle size is smaller.

#### 3.1.2. Effect of CaCO_3_ Particle Size and Content on Microscopic Morphology

**Figure 2:**
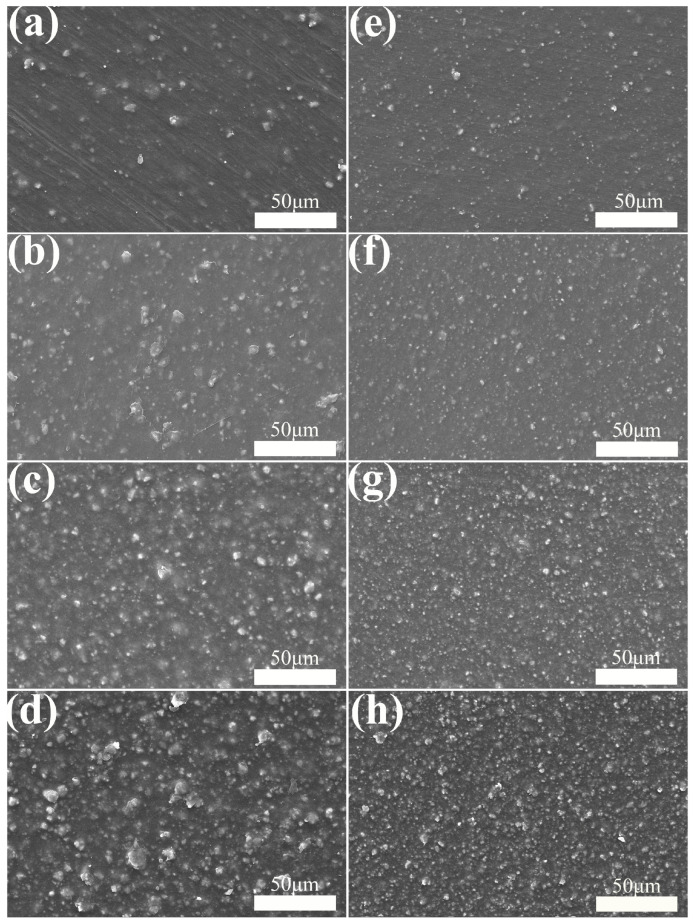
Effect of CaCO_3_ particle size and filling amount on the microscopic morphology of PBAT/CaCO_3_ composite films. (**a**–**d**) 1250 mesh CaCO_3_: (**a**) 9 wt%; (**b**) 18 wt%; (**c**) 27 wt%; (**d**) 36 wt%. (**e**–**h**) 2000 mesh CaCO_3_; (**e**) 9 wt%; (**f**) 18 wt%; (**g**) 27 wt%; (**h**) 36 wt%.

The effects of 1250 mesh and 2000 mesh unmodified CaCO_3_-filled PBAT on the microscopic morphology of the composite films are shown in Figure 2a–h.

Firstly, it can be seen from the figures that there is an obvious agglomeration of CaCO_3_ particles in the composite system, forming independent agglomerates, which indicates that the compatibility of CaCO_3_ particles and PBAT matrix is poor and the interfacial bonding is not good. When the film is subjected to external forces, it is easy to fracture from the interface, resulting in a significant decrease in the mechanical properties of the composite film. In addition, it can be seen from the comparison of Figure 2e–h that when the addition of CaCO_3_ is small, CaCO_3_ is not uniformly distributed in the composite system due to the agglomeration of particles. With the increase in CaCO_3_ content, CaCO_3_ particles are gradually distributed uniformly in the system, but the agglomeration phenomenon becomes more and more obvious. When the addition amount of CaCO_3_ is 27%, the distribution of CaCO_3_ particles in the system is relatively uniform, and the size of the agglomerates does not increase significantly.

When the same amount of CaCO_3_ particles is added for both sizes, as shown in Figure 2a,e, it can be seen that when the particle size is smaller, the agglomerates of CaCO_3_ particles are also smaller.

The results of the study on PBAT filled with unmodified CaCO_3_ show that the tensile properties of PBAT/CaCO_3_ composite films are severely degraded by PBAT filled with unmodified CaCO_3_ and cannot meet the market application standards. Therefore, surface modification of CaCO_3_ is necessary to increase the compatibility of CaCO_3_ with PBAT. The results of the study on the particle size and content of CaCO_3_ show that when using 2000 mesh CaCO_3_ with 27% addition, the CaCO_3_ particles are more uniformly distributed in the system, and the mechanical properties decrease less on the basis of reducing the production cost of the composite. Therefore, the formulation with 2000 mesh CaCO_3_ and 27% addition is chosen for the subsequent work for the modification study.

### 3.2. Effect of TC-Modified CaCO_3_ on Properties of PBAT/CaCO_3_ Composite Film

#### 3.2.1. FTIR Analysis of Modified CaCO_3_

**Figure 3:**
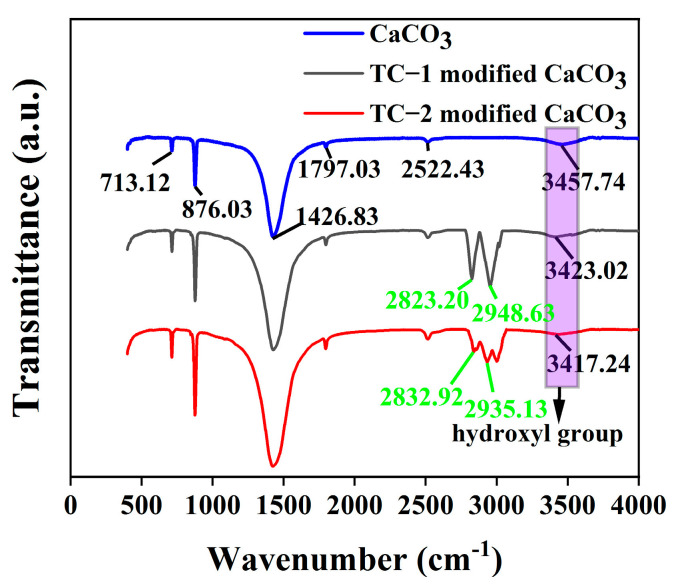
FTIR analysis spectra before and after CaCO_3_ modification.

To investigate the mechanism of TC-modified CaCO_3_, the changes of CaCO_3_ before and after modification are compared using Fourier infrared spectroscopy analysis, as shown in Figure 3.

As can be seen in Figure 3, the absorption peak at 3457 cm^−1^ is the stretching vibration peak of the hydroxyl group, which is the residual moisture in the CaCO_3_ sample. The weak absorption peak of the hydroxyl group is due to the fact that the CaCO_3_ is dried in an oven at 100 °C for 10 h before modification, and very little moisture remains. The absorption peak at 2522 cm^−1^ is the stretching vibration peak of the O=C=O group; the absorption peak at 1797 cm^−1^ is the stretching vibration peak of the C=O group; the absorption peak at 1426 cm^−1^ is the asymmetric stretching vibration peak of carbonate ion in CaCO_3_; the absorption peak at 876 cm^−1^ is the out-of-plane bending vibration peak of the carbonate ion, and the absorption peak at 713 cm^−1^ is the in-plane bending vibration peak of carbonate ion. The absorption peak at 876 cm^−1^ is the out-of-plane bending vibration peak of the carbonate ion, and the absorption peak at 713 cm^−1^ is the in-plane bending vibration peak of the carbonate ion [45].

From the results of FTIR analysis, it can be seen that the modified CaCO_3_ shows new absorption peaks near 2820 cm^−1^ and 2940 cm^−1^, which are the telescopic vibrational absorption peaks of -CH_3_- and -CH_2_- in the organic coupling agent, respectively, indicating that the coupling agent has successfully encapsulated on the surface of CaCO_3_ and formed new chemical bonds [46]. In addition, the increase in the transmittance of the hydroxyl absorption peak in the modified CaCO_3_ indicates that the absorbance of the hydroxyl group in the modified CaCO_3_ is weakened, the hydroxyl group is reduced, and the TC reacts with the hydroxyl group on the surface of CaCO_3_ to form a new chemical bond [47]. In addition, the absorption peaks of hydroxyl groups in TC-2-modified calcium carbonate diminish more obviously, indicating that the reaction between TC-2 and hydroxyl groups is more complete and the modification effect is better.

**Figure 4:**
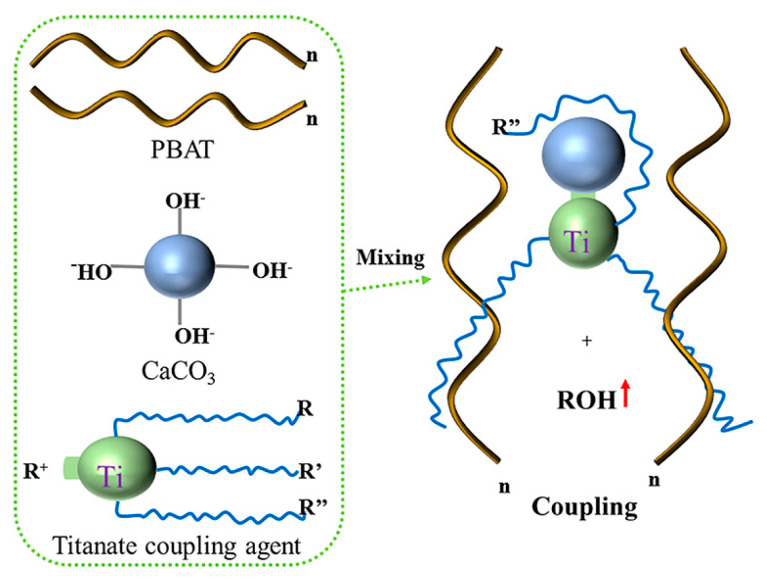
Mechanism of coupling action of titanate coupling agent.

Based on the FTIR test results, we determine the mechanism of action of the titanate coupling agent, as shown in Figure 4.

#### 3.2.2. Mechanical Properties

**Figure 5:**
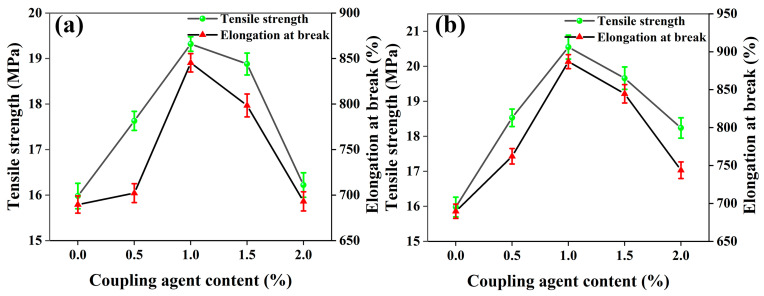
Effects of titanate coupling agent modified/CaCO_3_ on tensile properties of PBAT/CaCO_3_ films (**a**) TC-1 and (**b**) TC-2.

The effects of TC-1 and TC-2 additions on the tensile properties of PBAT/modified CaCO_3_ films are shown in Figure 5a,b.

As can be seen from the figure, the tensile properties of the composite films prepared from PBAT filled with both coupling agents modified CaCO_3_ show a trend of first increasing and then decreasing compared to the films prepared without modified CaCO_3_. The tensile strength of the films is the highest at 1% coupling agent addition, and the tensile properties of the composite films prepared by TC-1 modification reach 19.32 MPa, which is 17.29% higher than that of the films prepared without modified CaCO_3_. The tensile strength of the films prepared by TC-2 modification reaches 20.55 MPa, which is 28.60% higher than that of the films prepared without modified CaCO_3_. It can be seen that the tensile properties of the films are influenced by the type and content of the coupling agent. The amount of coupling agent added depends on the specific surface area of the treated object, and the larger the specific surface area, the more coupling agent is added [48]. Too little coupling agent addition will lead to an insignificant modification effect, and too much will lead to coupling agent cross-linking. In the mechanical property test, it can be seen that when the coupling agent addition is 0.5%–1%, the tensile property of the film is improved, and the modification effect is obvious. This is because the addition of a coupling agent prevents the agglomeration of CaCO_3_ particles, and the reduction in CaCO_3_ agglomerates reduces the tendency of crack initiation and expansion. When the coupling agent is added at 1.5%–2%, the tensile properties of the films show a decrease instead, which is due to the cross-linking of the coupling agent.

In addition, the properties test shows that when the content of TC is the same, such as P2-3/TC1-2 and P2-3/TC2-2, the mechanical properties of the composite film modified by TC-2 are better than those modified by TC-1. In the composite system, the interfacial bonding effect of the matrix phase and reinforcement phase and the dispersion degree of the reinforcement phase in the matrix phase directly affect the properties of the composite materials, especially the mechanical properties. Improving the interfacial adhesion between them can improve the performance of the composite film. For the purpose of this study, the mechanical properties of PBAT/CaCO_3_ films modified by TC-2 are higher, which may be because the pyrophosphate oxy group contained in the TC-2 structure plays a role in strengthening adhesion. In addition, TC-2’s long-carbon paraffin group is longer, and it is easier to entangle with the PBAT matrix, which improves the interface bonding force.

#### 3.2.3. Micromorphological Analysis

**Figure 6:**
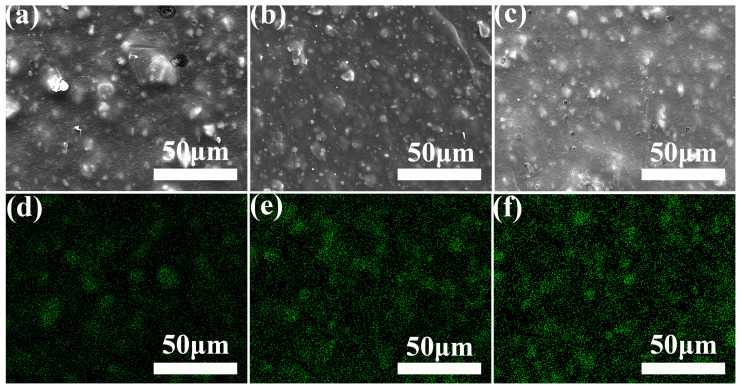
Effect of titanate coupling agent modified CaCO_3_ on microstructure and calcium distribution of PBAT/CaCO_3_ composite film. (**a**,**d**) no modification. (**b**,**e**) TC-1 modification. (**c**,**f**) TC-2 modification.

The effects of two titanate coupling agents modified with CaCO_3_ on the microscopic morphology and calcium element distribution of PBAT/CaCO_3_ films are observed by scanning electron microscopy and EDS energy spectrum analysis, as shown in Figure 6a–f.

Figure 6a–c shows the surface microscopic morphology of the films before and after TC modification. It can be seen from the figures that the number and size of CaCO_3_ agglomerates in the titanate coupling agent-modified composite film system are significantly reduced, and the surface of the TC-2-modified film is smoother than that of the TC-1-modified film, which further confirms the conclusion of the tensile properties discussed in Section 3.2.2. In addition, we have analyzed the distribution of calcium elements in the composite film system before and after TC modification by EDS, as shown in Figure 6d–f. It can be seen from the figures that the modified CaCO_3_ is more uniformly distributed in the composite system. The comparison of Figure 6e,f shows that the CaCO_3_ is more uniformly distributed in the composite system after TC-2 modification compared to TC-1 modification.

#### 3.2.4. Melting and Crystallization Behavior

**Figure 7:**
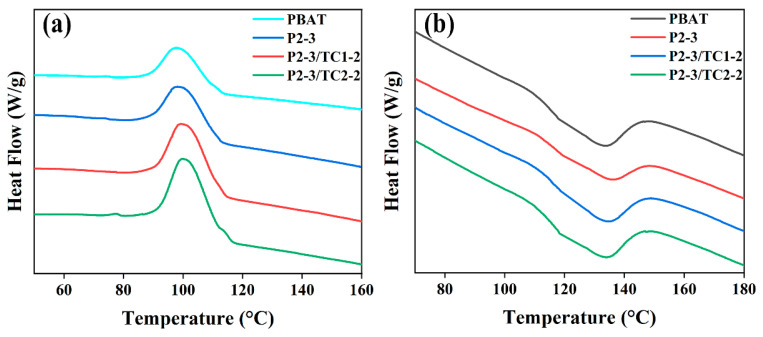
DSC curves of PBAT and PBAT/CaCO_3_ composite films. (**a**) crystallization curve. (**b**) melting curve.

The DSC curves of pure PBAT and PBAT/CaCO_3_ composite films are analyzed by differential scanning calorimetry, as shown in Figure 7a,b. The crystallization parameters of pure PBAT and PBAT/CaCO_3_ composites are shown in Table 3.

From Figure 7a,b and Table 3, we can see that the T_c_, T_m_, and H_m_ of the composites increase after the addition of CaCO_3_ compared with the pure PBAT, and the X_c_ also increases accordingly. The X_c_ of P2-3/TC2-2 is the highest, from 7.09% to 14.83%. This is mainly due to the fact that the addition of CaCO_3_ acts as a heterogeneous nucleation and promotes crystallization growth, which makes PBAT easier to form crystals by regular arrangement and enhances the crystallization ability [49].

#### 3.2.5. Thermal Stability

**Figure 8:**
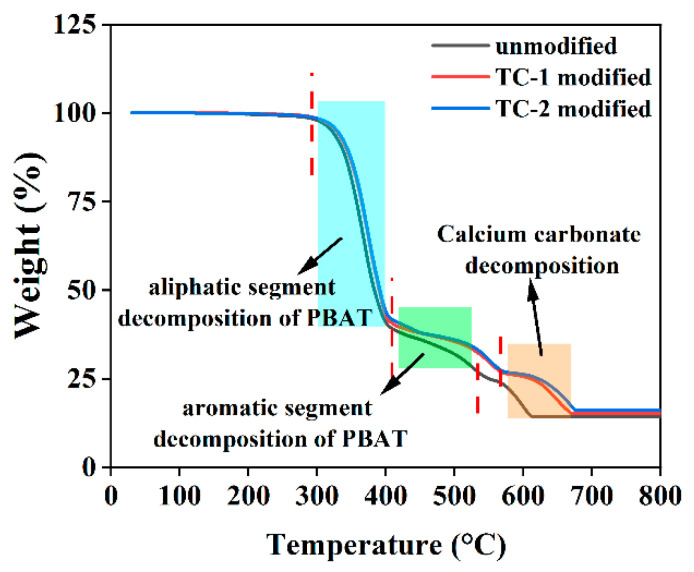
Effect of titanate coupling agent-modified CaCO_3_ on thermal stability of PBAT/CaCO_3_ composite films.

The effect of titanate coupling agent-modified CaCO_3_ on the thermal stability of PBAT/CaCO_3_ composite films is tested using a comprehensive thermal analyzer, as shown in Figure 8.

PBAT is a random co-polyester synthesized by aliphatic and aromatic, and the decomposition temperature of aliphatic and aromatic segments is different, so the decomposition process of PBAT is divided into two parts. From 300 °C to 400 °C, the mass of three groups of samples decreased to 40%, which is caused by the aliphatic decomposition of PBAT. The temperature at which the aliphatic segments of the three groups of films began to decompose is similar, indicating that TC has little effect on the decomposition of PBAT aliphatic segments. From 400 °C to 530 °C, the quality of the three groups of samples continues to decline, and this stage is the decomposition process of aromatic segments. The results show that the decomposition rate of aromatic segments decreases after adding TC, which indicates that the addition of TC inhibits the decomposition of aromatic segments. After 530 °C, the quality of the sample further decreases, which is a process of thermal decomposition of CaCO_3_. After adding TC, the decomposition temperature of CaCO_3_ increases from 533.9 °C to 566.1 °C, which indicates that the introduction of TC inhibits the decomposition of CaCO_3_ and improves the thermal stability of the film [50]. In addition, the TG results show that the films modified by TC-2 have a more obvious inhibition effect on the decomposition of aromatic segments and CaCO_3_ because the molecular structure of TC-2 contains pyrophosphate oxy group, which has a certain flame-retardant effect [50].

#### 3.2.6. Hydrophilicity

**Figure 9:**
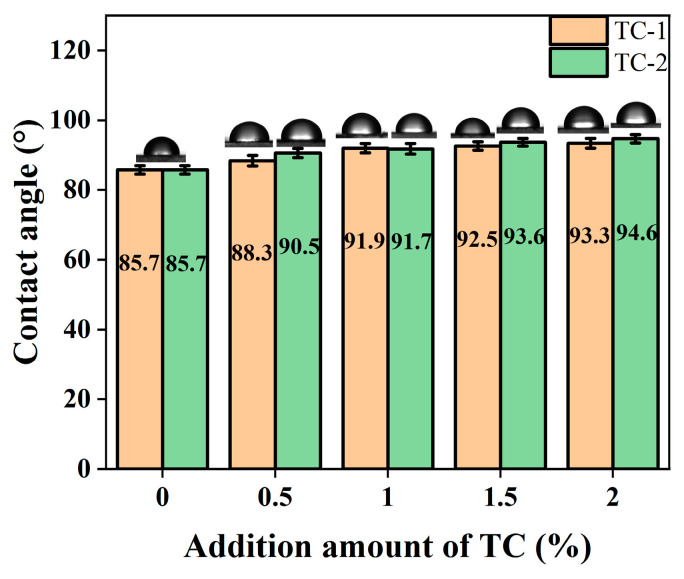
Effect of TC-modified CaCO_3_ on contact angle of PBAT/CaCO_3_ composite films.

The effect of two titanate coupling agents modified with CaCO_3_ on the contact angle of composite PBAT/CaCO_3_ films is tested with a contact angle measuring instrument and image processing software, as shown in Figure 9.

The contact angle measurement results show that the contact angle of the unmodified CaCO_3_-filled PBAT composite film is the smallest at 85.7°, which is due to the presence of the -OH groups on the surface of CaCO_3_, a hydrophilic component. The contact angle of the TC-modified CaCO_3_-filled PBAT composite film is larger than that of the unmodified CaCO_3_-filled PBAT composite film, and the contact angle of the film gradually increases with the addition of the coupling agent. When the content of the two coupling agents is 2%, the contact angles of the films reach the maximum of 93.3° and 94.6°, respectively, which indicates that the addition of titanate coupling agent made the composite films more hydrophobic. The reason for this is that the short carbon chain alkoxy groups in the coupling agent react with the hydrophilic groups on the surface of CaCO_3_, resulting in a reduction in hydrophilic groups in the composite and, thus, an increase in hydrophobicity [51]. In addition, the comparison between TC-1 and TC-2 in Figure 9 shows that the contact angle of the composite film prepared by TC-2 modified CaCO_3_ is larger. According to the results of FTIR analysis, TC-2 reacts more completely with the hydroxyl groups on the surface of CaCO_3_, and the material exhibits better hydrophobicity.

#### 3.2.7. Water Absorption

**Figure 10:**
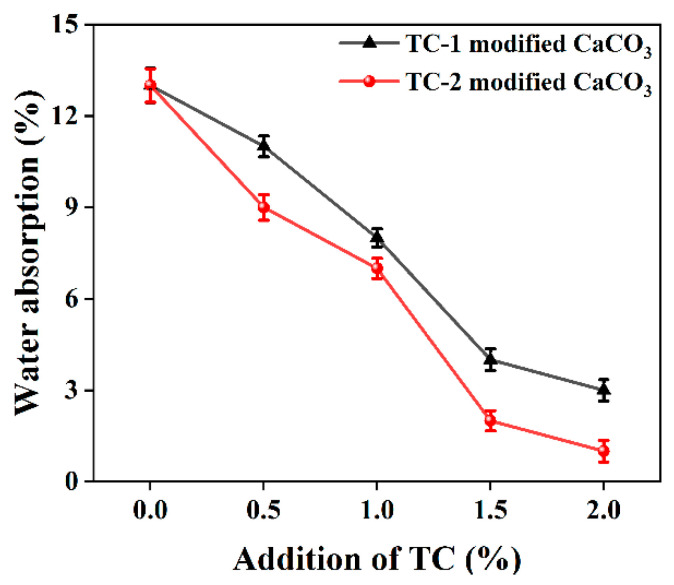
Effect of TC-modified CaCO_3_ on water absorption of PBAT/CaCO_3_ films.

The effects of unmodified CaCO_3_ and two titanate coupling agents modified with CaCO_3_ on the water absorption of PBAT/CaCO_3_ composite films were tested according to the international standard ISO 62, as shown in Figure 10.

The water absorption test results show that the water absorption of the composite films prepared by TC-modified CaCO_3_ is less than that of the composite films prepared by unmodified CaCO_3_. Moreover, the water absorption of the films gradually decreases from 13% to less than 5% as the amount of coupling agent added gradually increases from 0% to 2%. When the additional amount of TC-2 is 2%, the water absorption of the films is only 1%, which indicates that the addition of a coupling agent makes the composite films more hydrophobic, which is basically consistent with the conclusion obtained from the contact angle measurement.

For PBAT-degradable plastic packaging products, it is very important to reduce their water absorption and improve their hydrophobicity [42] because the water absorption of products will accelerate the degradation of PBAT, which will lead to the adhesion of plastic packaging films. In addition, the degradation of PBAT will lead to the rapid decline of the mechanical properties of plastic packaging products, thus greatly reducing the service life of packaging films. Due to the addition of TC, the hydrophobicity of the composite film is significantly improved, so the composite film prepared in this study can be used as a waterproof material for biodegradable plastics [52].

#### 3.2.8. Water Vapor Transmission Performance

The water vapor transmission properties of pure PBAT and PBAT/CaCO_3_ composites are tested using a water vapor transmission tester, as shown in Table 4.

As can be seen from Table 4, the WVTR of pure PBAT is 600.37 g/(m^2^·24 h), and the WVP is 3.45 × 10^−13^ g·cm/(Pa·s·cm^2^). The WVTR and WVP of the composites are lower than those of pure PBAT after the CaCO_3_ filling modification. Among them, the TC-2 modified composites exhibit lower WVTR and WVP than those of pure PBAT. The decrease in the water vapor transmission performance of the material is mainly due to the fact that the CaCO_3_ filler modification is dispersed in the PBAT matrix, forming a barrier layer, and the path of water vapor transmission through the composite is curved, which reduces the probability of water vapor transmission of the composite and improves the water vapor transmission performance of the composite [53]. The modified CaCO_3_ filling is more uniformly dispersed in the PBAT matrix and forms more barrier layers; thus, the WVTR and WVP of the composites decrease more significantly.

## 4. Conclusions

To investigate the effects of CaCO_3_ particle size (1250 mesh, 2000 mesh), particle content (0–36%), and titanate coupling agent (TC) surface modification on PBAT/CaCO_3_ films, the films were prepared by the twin-screw extruder and single-screw extrusion blow-molding machine. The results showed that the size and content of CaCO_3_ particles significantly affected the tensile properties of the composites. The addition of unmodified CaCO_3_ decreased the tensile properties of the composites by more than 30%. TC-modified CaCO_3_ had a significant effect on the tensile properties, crystallization, melting behavior, thermal stability, water contact angle, water absorption, water vapor transmission rate, and microscopic morphology of PBAT/CaCO_3_ composite films.

The results of tensile properties showed that the addition of TC caused the tensile strength and elongation at the break of the composite films to increase and then decrease, and the performance of PBAT/modified CaCO_3_ composite films was better than that of PBAT/unmodified CaCO_3_ films. When titanate coupling agent 201 (TC-2) was added at 1%, the tensile strength of the films reached 20.55 MPa, which exceeded that of pure PBAT and increased by 28.60% compared with PBAT/unmodified CaCO_3_ films.

Microscopic morphological analysis showed that the unmodified CaCO_3_ was significantly agglomerated in the composite system and had poor interfacial bonding with the PBAT matrix. The addition of a titanate coupling agent effectively improved the interface between CaCO_3_ and PBAT; the agglomerates of CaCO_3_ were significantly reduced, and the surface of the material was smoother. Energy dispersive spectrometer mapping analysis showed that the modified CaCO_3_ was more uniformly distributed in the system. The stronger interfacial bonding effect and good dispersion were the reasons for the improved tensile properties of PBAT/modified CaCO_3_ composite films.

The DSC test results showed that the addition of unmodified CaCO_3_ improved the melting temperature, crystallization temperature, and crystallinity of the composite films, and the addition of TC further improved the crystallization temperature and crystallinity of the composite films. Among them, when TC-2 was added at 1%, the composite film had the highest melting enthalpy and the highest crystallinity of 14.83%, which exceeded that of pure PBAT and PBAT/unmodified CaCO_3_ composite films. TG analysis showed that the addition of a titanate coupling agent improved the thermal stability of the material, and the decomposition temperature of TC-2 modified CaCO_3_ increased from 546.5 °C to 566.1 °C.

The results of contact angle and water absorption tests showed that TC improved the hydrophobicity of the composite films by reacting with the hydrophilic hydroxyl groups on the surface of CaCO_3_. With the increase in TC addition from 0% to 2%, the contact angle of the composite films prepared from TC-1 and TC-2-modified CaCO_3_ increased from 85.7° to 93.3° and 94.6°, respectively, and the water absorption decreased from 13% to 3% and 1%, respectively. The results of the water vapor transmission performance tests showed that the water vapor barrier performance of the composites was enhanced by the modified CaCO_3_ filling. Among them, the WVTR of the material was reduced by 27.99%, and the corresponding WVP was reduced by 43.19% for the addition of TC-2 at 1% compared to pure PBAT.

Finally, this study confirms that the PBAT/modified CaCO_3_ composite film has a very good overall performance. This low-cost, high-performance, fully degradable composite film has a broad application prospect in the field of plastic packaging.

## Figures and Tables

**Table 1:** Experimental formula of PBAT film filled with CaCO_3_ with different particle sizes and contents.

Samples	PBAT (wt%)	CaCO_3_ (wt%)	
		1250 Mesh	2000 Mesh
PBAT	100	-	-
P1-1	91	9	
P1-2	82	18	
P1-3	73	27	
P1-4	64	36	
P2-1	91		9
P2-2	82		18
P2-3	73		27
P2-4	64		36

**Table 2:** Experimental formula of PBAT film filled with CaCO_3_ modified by two titanate coupling agents.

Samples	PBAT (wt%)	CaCO_3_ (wt%)	TC-1 (wt%)	TC-2 (wt%)
P2-3	73	27	-	-
P2-3/TC1-1	73	27	0.5	
P2-3/TC1-2	73	27	1	
P2-3/TC1-3	73	27	1.5	
P2-3/TC1-4	73	27	2	
P2-3/TC2-1	73	27		0.5
P2-3/TC2-2	73	27		1
P2-3/TC2-3	73	27		1.5
P2-3/TC2-4	73	27		2

**Table 3:** Crystallization parameters of PBAT and PBAT/CaCO_3_ composite films.

Samples	T_c_ (°C)	T_m_ (°C)	ΔH_m_ (J/g)	X_c_ (%)
PBAT	97.51	133.49	8.08	7.09
P2-3	97.82	136.21	11.20	13.46
P2-3/TC1-2	99.32	134.82	12.14	14.59
P2-3/TC2-2	99.67	133.79	12.34	14.83

Note: T_c_ is the crystallization temperature; T_m_ is the melting temperature; ΔH_m_ is the enthalpy of melting; X_c_ is the degree of crystallinity.

**Table 4:** Water vapor permeability of PBAT and PBAT/CaCO_3_ composite films.

Samples	WVTR [g/(m^2^·24 h)]	WVP [g·cm/(Pa·s·cm^2^)]
PBAT	600.37	3.45 × 10^−13^
P2-3	549.10	2.39 × 10^−13^
P2-3/TC1-2	487.82	2.06 × 10^−13^
P2-3/TC2-2	432.30	1.96 × 10^−13^

## Data Availability

The data used in this research have been properly cited and reported in the main text.
